# Effects of Attachment Placement on Palatal Root Torque Control of Maxillary Incisors with Clear Aligners: A Finite Element Study

**DOI:** 10.3390/jcm15083111

**Published:** 2026-04-19

**Authors:** Youn-Kyung Choi, Soon-Pill Jeong, Sung-Hun Kim, Seong-Sik Kim, Yong-Il Kim

**Affiliations:** 1 Department of Orthodontics, Dental Research Institute, School of Dentistry, Pusan National University, Yangsan 50612, Republic of Korea; youngyng@pusan.ac.kr (Y.-K.C.); siesta@pusan.ac.kr (S.-H.K.); softid@pusan.ac.kr (S.-S.K.); 2 Dental and Life Science Institute, School of Dentistry, Pusan National University, Yangsan 50612, Republic of Korea; 3 YEBA Orthodontic Clinic, Busan 47245, Republic of Korea; spjeong0707@gmail.com

**Keywords:** attachment, clear aligners, finite element analysis, torque

## Abstract

**Objective:** The objective of this study is to evaluate the biomechanical effects of different attachment placement strategies using rectangular attachments on palatal root torque control of maxillary central and lateral incisors with clear aligners. **Methods:** Three-dimensional finite element analysis was performed to simulate simultaneous 1° palatal root torque of maxillary central and lateral incisors. Six attachment configurations were evaluated: no attachment (control), canine-only, both incisors, central incisor-only, lateral incisor-only, and all anterior teeth. Three-dimensional tooth displacement and torque expression were analyzed across 200 iterative simulations. Model validation was confirmed through mesh convergence analysis and comparison with published studies. **Results:** Only the control and canine-only groups simultaneously achieved the appropriate torque direction for both incisors. Attachments on central incisors produced reverse torque, with the central incisor-only group showing the most severe magnitude, while the control and canine-only groups achieved expected directions, validating model reliability. Lateral incisors exhibited different responses, including reverse torque in the lateral incisor-only group. The canine-only attachment demonstrated the most balanced torque expression. Increasing anterior attachments was associated with greater extrusion and canine displacement. **Conclusions:** Attachment placement using rectangular attachments significantly influenced torque expression during palatal root torque. Central and lateral incisors responded differently to attachments, and certain configurations produced reverse torque. For small torque movements (1–2°), a “less is more” approach using rectangular canine attachments for anchorage proved most effective, suggesting that anchorage may be more critical than incisor attachments for anterior torque control.

## 1. Introduction

Advances in digital technology and materials science have expanded the indications for clear aligners (CAs) from mild malocclusions to complex cases, including extractions [1,2]. However, as their use has broadened, biomechanical limitations have become more evident, and the predictability of complex tooth movements remains inferior to that of fixed appliances [3,4]. Systematic reviews consistently report that anterior extrusion, the rotation of teeth with round crowns, and root torque control are among the most challenging movements to achieve with CAs [3,5].

Among these movements, palatal root torque of the anterior teeth is considered particularly challenging [6,7], with clinical studies showing that only 35–49% torque achievement is typically obtained in clinical CA therapy [8,9,10]. Early clinical observations similarly demonstrated limited incisor root control with the Invisalign system, highlighting that torque predictability has remained a persistent challenge since the early adoption of CA therapy [11]. This limited predictability is largely attributed to the flexibility of CA materials and constraints imposed by crown morphology, which often result in uncontrolled tipping rather than the intended torque [6,12]. In fixed appliances, torque is expressed through three-dimensional wire–slot interactions between rectangular archwires and bracket slots, whereas CAs require a force couple acting in opposite directions at the incisal edge and cervical region [6]. However, because CAs are fabricated from materials with a low elastic modulus and relatively weak cervical stiffness, they are prone to losing contact at the palatal incisal edge, which is essential for torque generation [6,12,13]. Consequently, CAs tend to produce uncontrolled labial tipping instead of effective palatal root torque [14,15].

To address these limitations, attachments have been introduced to improve retention and enhance three-dimensional tooth control [3,4]. Simon et al. [10] reported that anterior torque accuracy increased from 35.2% without attachments to 49.1% with attachments. Recent finite element and in vitro studies further indicate that attachment shape, position, and placement configuration significantly influence torque control, with the greatest torque moments observed when horizontal ellipsoid attachments are positioned in the middle third of the crown [14,16,17]. Furthermore, Elshazly et al. demonstrated that attachment configuration and trim line design jointly influence the aligner force system, with extended trim lines and rectangular attachments producing greater force magnitudes and improved stress distribution within the PDL [18].

However, existing evidence remains limited in several aspects. Most studies have evaluated the torque control of individual teeth rather than comparing central and lateral incisors simultaneously under identical conditions. For example, Hong et al. [14] investigated various auxiliaries during a 1° palatal root torque of the maxillary central incisor and found that semi-ellipsoid attachments improved labiolingual tipping control, whereas power ridges provided superior root control but were associated with distal torsion. Similarly, Karsli et al. [15] focused exclusively on lateral incisors, assessed attachment effects during labial movement of palatally positioned lateral incisors, and reported that attachments on both labial and palatal surfaces demonstrated the least tipping and displacement. Since central and lateral incisors differ in root morphology, periodontal ligament (PDL) surface area, and crown-to-root ratio [19,20,21], their biomechanical responses and dependence on attachments may also differ. While prior FEA studies have focused on individual incisor torque mechanics [6,12,14,15] or specific auxiliary designs [13,16], a direct comparison of how different attachment placement strategies—including canine-only configurations—differentially affect both central and lateral incisors under identical torque conditions remains limited, complicating the formulation of tooth-specific clinical guidelines.

Additionally, the anchorage contribution of canine attachments during anterior torque movement has not been systematically evaluated [6,21], and few studies have compared attachment strategies across the full spectrum—from no attachments to attachments on all anterior teeth—making it difficult to establish evidence-based clinical guidelines for optimal attachment placement. Understanding the biomechanical effects of different attachment configurations, including canine-only anchorage versus direct incisor attachments, is essential for improving torque predictability in CA treatment.

Therefore, this study systematically evaluated the effects of different attachment placement strategies on palatal root torque control of the maxillary anterior teeth using three-dimensional finite element analysis (FEA). This study tested the following hypotheses: (1) attachments improve torque control compared to no attachments; (2) the lateral incisor shows greater dependence on attachments than the central incisor due to its smaller root and lower PDL surface area; and (3) canine attachments enhance anchorage during anterior torque movement. Six scenarios, ranging from no attachments to attachments on all anterior teeth, were compared to provide evidence-based guidance for tooth-specific attachment design in CA treatment. The null hypothesis was that attachment placement would not influence the palatal root torque expression of maxillary incisors during clear aligner treatment.

## 2. Materials and Methods

A three-dimensional maxillary model including the dentition, alveolar bone, and periodontal ligament (PDL) was generated from cone-beam computed tomography data and segmented using 3D Slicer open-source software (ver. 5.10.0, www.slicer.org). The tooth positions were adjusted to represent normal alignment, and the finite element geometry was subsequently prepared for simulation. CAs were digitally designed and modeled using SOLIDWORKS 2024 (Dassault Systèmes, Vélizy-Villacoublay, France). Both the maxillary central and lateral incisors were programmed with 1° palatal root torque as the target position, with the incisal edge of the central incisor defined as the center of rotation. A torque magnitude of 1° was selected to represent the small, single-step torque movement commonly used in CA staging, enabling the evaluation of early biomechanical responses to attachment placement. This approach allows investigation of the initial biomechanical effects of attachment placement while minimizing confounding factors associated with larger cumulative movements. The CAs were modeled with a uniform thickness of 0.5 mm from the crown surface according to each experimental design, and the trimming line followed a scalloped design at the gingival margin level.

**Figure 1:**
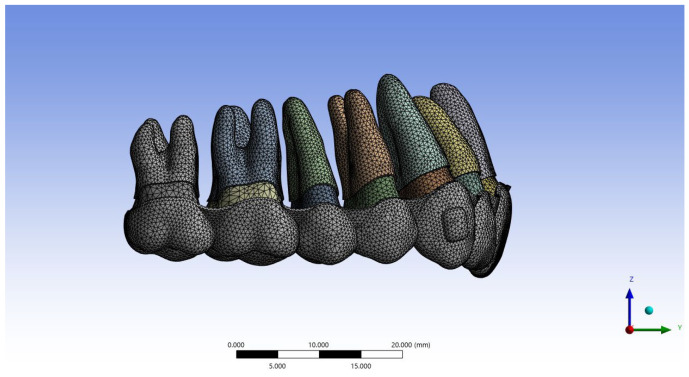
Finite element model of the clear aligner and dentition.

Attachments were designed as vertical rectangular shapes on the maxillary canines and horizontal rectangular shapes on the maxillary central and lateral incisors. All attachments were positioned on the labial surface in the middle third of the crown (Figure 1).

**Figure 2:**
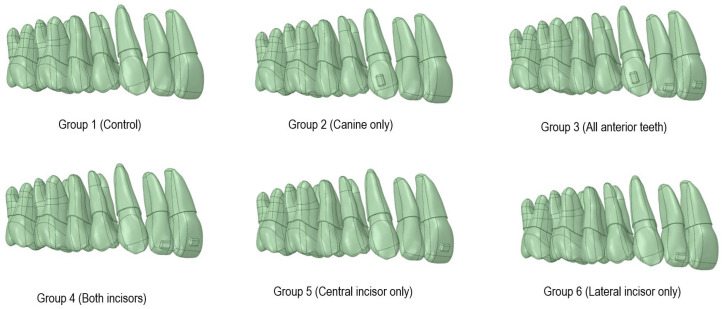
Six experimental groups.

To systematically evaluate the influence of attachment placement on torque control, six experimental groups were established, ranging from no attachments to attachments on all anterior teeth (Figure 2):

1. Group 1 (Control): No attachments on any teeth;
2. Group 2 (C only): Attachment on the maxillary canine (#3) only;
3. Group 3 (All anterior): Attachments on the central incisor, lateral incisor, and canine (#1, #2, and #3);
4. Group 4 (Both incisors): Attachments on both central and lateral incisors (#1 and #2);
5. Group 5 (CI only): Attachment on the maxillary central incisor (#1) only;
6. Group 6 (LI only): Attachment on the maxillary lateral incisor (#2) only.

The maxillary canine was designated as the anchorage tooth to assess the anchorage response during anterior palatal root torque.

All components were assumed to be homogeneous, linear, and made of elastic materials, with mechanical properties adopted from previous studies (Table 1) [22]. The tooth structure was modeled as a homogeneous material rather than separating enamel and dentin to simplify the computational model, as periodontal ligament behavior plays a dominant role in orthodontic tooth displacement. Similarly, the alveolar bone was modeled as a homogeneous structure rather than separating cortical bone and lamina dura, which is a common simplification step in orthodontic FEA to reduce model complexity while maintaining reliable simulation of tooth movement. FEA modeling and analysis were performed using ANSYS 2024R (ANSYS Inc., Canonsburg, PA, USA).

Contact conditions were defined as follows: the tooth–PDL interface and tooth–attachment interfaces were modeled as bonded contacts; the tooth–tooth interface was modeled as a frictionless contact, and the CA–tooth and CA–attachment surfaces were modeled as frictional contacts with a coefficient of friction of μ = 0.2 [23]. To prevent rigid-body motion during the simulation, the basal surface of the alveolar bone was fixed in all directions, while deformation of the teeth and periodontal ligament was allowed under the applied aligner forces.

A global coordinate system was used to define directions in three-dimensional space: the *X*-axis represents the transverse direction (positive toward the right); the *Y*-axis represents the anteroposterior direction (positive toward the anterior), and the *Z*-axis represents the vertical direction (positive toward the gingiva/intrusion).

Tooth movement simulation was performed for 200 iterative cycles based on the method reported by Yokoi et al. [24]. Progressive displacement of the teeth and PDL resulting from alveolar bone remodeling was calculated through FEA, and the outer surface of the PDL adjacent to the alveolar socket was displaced by the amount of initial movement produced by elastic deformation of the PDL. The number of iterations was selected to ensure sufficient accumulation of rotational displacement during the simulation process. In the present study, which focused on torque expression rather than translational movement, a higher number of iterations (200 cycles) was required compared to studies evaluating bodily or tipping movements, as rotational displacement accumulates more gradually and requires additional cycles to reach a stable state. The number of iterations (N) represents the number of simulation cycles after CA placement and reflects computational stages rather than the actual clinical time.

### 2.1. Model Validation

The reliability of the FEA model was verified through several validation procedures. Mesh convergence analysis was performed by progressively refining the mesh density until displacement values changed by less than 2% between successive iterations. The final model consisted of approximately 850,000 elements. Displacement patterns in the control group (Group 1) were compared with values reported by Yokoi et al. [24] for similar torque movements, showing agreement within 15%.

Internal validation was provided by the achievement of expected torque directions in multiple groups. Groups 1, 2, and 6 produced torque in the planned direction for central incisors (75.0–105.4% achievement), and Groups 1, 2, and 5 did so for lateral incisors (47.1–76.2% achievement). The occurrence of reverse torque only in specific configurations (Groups 3, 4, and 5 for central incisors; Groups 3, 4, and 6 for lateral incisors) represents configuration-specific biomechanical phenomena rather than systematic computational errors.

### 2.2. Outcome Variables

The following outcome variables were measured and compared across the six groups.

#### 2.2.1. Coronal and Radicular Displacement

Three-dimensional displacement of the incisal edge (or cusp tip) and root apex of the central incisors, lateral incisors, and canines was measured along the X-, Y-, and Z-axes. The center of rotation was estimated based on the relative displacement between the crown and root apex obtained from the finite element simulation, allowing interpretation of the rotational pattern of each tooth during torque expression.

#### 2.2.2. Torque Expression Achievement

Angular changes in the tooth long axis in the sagittal plane were measured. The torque achievement rate relative to the planned 1° palatal root torque was calculated as Torque achievement rate (%) = (Actual rotation angle/Target 1°) × 100.

## 3. Results

### 3.1. Tooth Displacement Patterns

When 1° palatal root torque was applied simultaneously to the maxillary central and lateral incisors, distinct three-dimensional displacement patterns were observed across the six attachment groups. The displacement responses of the incisors and the anchorage canine are presented in Table 2 and Table 3, allowing a comparison of how different teeth responded to the same attachment configurations.

#### 3.1.1. Central Incisor Displacement

The central incisors exhibited marked intergroup differences, particularly in anteroposterior (*Y*-axis) displacement (Table 2). Root apex displacement showed two opposing patterns: Groups 1, 2, and 6 demonstrated palatal displacement in the planned direction, whereas Groups 4 and 5 displayed labial displacement opposite to the plan. Among these, Group 5 showed the greatest labial root apex displacement (+0.405 mm), while Group 2 demonstrated the most pronounced palatal displacement (−0.305 mm). Group 3 showed minimal displacement. Crown displacement followed a complementary pattern, with Groups 1, 2, and 6 showing labial displacement and Groups 3 and 4 exhibiting palatal crown displacement. Group 5 exhibited minimal crown displacement (+0.002 mm), indicating an abnormal shift in the center of rotation. Vertically, incisal edge extrusion occurred in most groups, with Group 1 showing the least (−0.009 mm) and Group 4 exhibiting the greatest (−0.204 mm).

#### 3.1.2. Lateral Incisor Displacement

The lateral incisors demonstrated different response patterns from the central incisors (Table 2). Root apex displacement was palatal in Groups 1, 2, and 5, consistent with the planned direction, with Group 2 showing the greatest palatal displacement (−0.242 mm). Groups 4 and 6 showed labial displacement opposite to the plan, while Group 3 showed minimal displacement. For crown displacement, Groups 1 and 2 showed labial movement, while Groups 3, 4, 5, and 6 exhibited palatal crown displacement, with Group 4 demonstrating the greatest magnitude (−0.107 mm). Vertical extrusion was observed in all groups, with Group 1 showing the least (−0.010 mm) and Group 4 exhibiting the greatest (−0.184 mm).

#### 3.1.3. Anchorage (Canine) Response

The maxillary canine, designated as the anchorage tooth, exhibited variable displacement across groups (Table 3). Root apex displacement occurred labially in all groups, ranging from +0.015 mm (Group 1) to +0.201 mm (Group 3). Tip displacement differed by group: Groups 1 and 2 showed labial displacement (+0.032 to +0.054 mm), whereas Groups 3, 4, 5, and 6 displaced palatally (−0.004 to −0.044 mm).

### 3.2. Torque Expression Comparison

**Figure 3:**
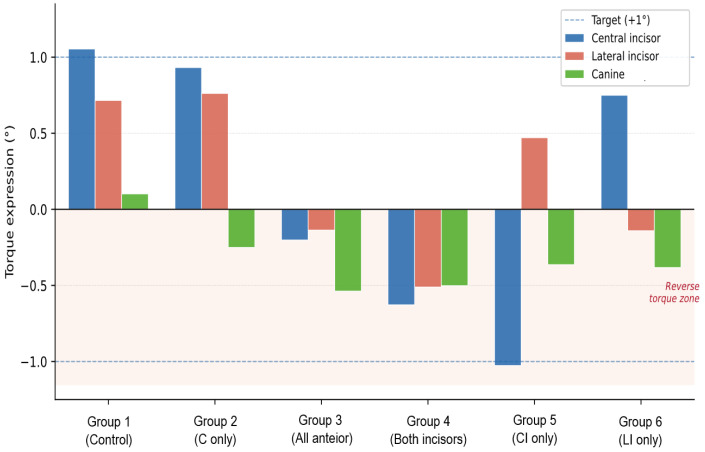
Torque expression of the maxillary incisors and canine across six attachment configurations.

When 1° palatal root torque was planned for both incisors, the achieved rotation angles varied significantly among groups, with reverse torque observed in several configurations (Table 4). Reverse torque was defined as angular rotation in the direction opposite to the intended palatal root torque. Overall, Groups 1 and 2 demonstrated the most favorable palatal root torque expression, whereas several configurations with incisor attachments produced reverse torque. For lateral incisors, Groups 1, 2, and 5 rotated in the planned direction, with Group 2 showing the highest torque achievement, whereas Groups 3, 4, and 6 exhibited reverse torque, with the most severe reverse torque observed in Group 4. Although torque was not planned for the canines, unwanted rotation occurred in all groups, with the greatest rotation observed in Group 3. Detailed angular values are presented in Table 4 and Figure 3.

### 3.3. Simultaneous Torque Achievement for Both Incisors

Only Groups 1 and 2 simultaneously achieved the intended torque direction for both central and lateral incisors. Group 2 demonstrated the most balanced torque achievement, whereas configurations with direct incisor attachments frequently resulted in reverse torque. The most severe reverse torque occurred in the central incisor-only configuration. Detailed torque achievement rates are presented in Table 4.

## 4. Discussion

This study evaluated the effects of attachment placement strategies on palatal root torque control of maxillary anterior teeth using FEA. Contrary to our first hypothesis, the presence of attachments did not consistently improve torque control, and certain configurations even induced reverse torque. Therefore, Hypothesis 1 was not supported by the results of this study. These findings challenge the conventional assumption that increasing the number of attachments necessarily improves biomechanical control in clear aligner therapy.

The most important finding was that the groups with central incisor attachments (Groups 3, 4, and 5) exhibited reverse torque, showing labial root movement instead of the planned palatal root torque. In contrast, only the control and canine-only attachment configurations produced torque in the intended direction for both incisors. This finding highlights the importance of anchorage support and suggests that increasing the number of incisor attachments does not necessarily improve torque control.

The severe reverse torque observed in the central incisor attachment configuration may appear atypical; however, it is best interpreted as a configuration-specific biomechanical response rather than a systematic computational artifact. The displacement pattern demonstrated labial root movement with minimal crown displacement, indicating a shift in the center of rotation and resulting in rotation opposite to the intended palatal root torque. However, the magnitude of reverse torque observed in this simulation should be interpreted as a directional biomechanical tendency rather than a direct clinical prediction. Clinically, such paradoxical responses may contribute to the well-documented limited predictability of anterior torque with CAs, where only 35–49% of the planned torque is typically achieved [8,9,10].

These findings differ from the general assumption that attachments necessarily improve torque control [3,5,25]. Simon et al. [10] reported improved anterior torque accuracy with attachments (49.1%) versus no attachment (35.2%), while our control group demonstrated higher torque expression. This discrepancy may be related to the limited torque magnitude that was evaluated (1°). These findings have significant clinical implications. First, for small torque movements (1–2°), a directional torque expression may be achievable without attachments. Second, the canine-only configuration yielded the most balanced torque achievement, suggesting that anchorage support can be more influential than incisor attachments. Third, indiscriminate use of central incisor attachments may produce adverse effects, and clinicians should reconsider the assumption that increasing the number of attachments necessarily improves outcomes.

The reverse torque phenomenon can be explained by a disrupted force-couple balance. Effective torque control requires a force couple formed by opposing forces at the palatal incisal edge and labial cervical region [6,12]. CAs fabricated from materials with a low elastic modulus are prone to losing contact at the palatal incisal edge [6,13], often resulting in uncontrolled labial tipping. When attachments were placed on the labial surface of the central incisor, the force-couple balance was disrupted. Attachments increase local stiffness and generate excessive labial crown force, producing an unfavorable moment system that tips the crown palatally while displacing the root labially [14,26]. This mechanism is supported by the marked labial root apex displacement (+0.405 mm) in Group 5, where crown displacement was minimal (+0.002 mm). When a labial attachment was placed on the central incisor, local stiffness increased dramatically, causing the aligner to apply excessive labial force at the attachment site. This force overwhelmed the palatal force at the incisal edge, which was already weakened by the low elastic modulus of the aligner material and the tendency to lose contact at the palatal incisal edge [6,13]. The resulting force imbalance created an unfavorable moment that tipped the crown palatally while displacing the root labially—the opposite of the intended torque.

Hong et al. [14] reported that palatal root torque can shift the center of rotation toward the root, leading to labial crown tipping. Our findings suggest that labial attachments may exacerbate this tendency. Cheng et al. [2] reported that moment-to-force ratio variations influence the location of the center of rotation and that the reverse torque in groups with central incisor attachments appears to reflect an unfavorable moment-to-force ratio that abnormally shifts the center of rotation. Ahmed et al. [26] demonstrated that the labial attachment position significantly affects force distribution and can produce adverse moment systems during incisor retraction, supporting the plausibility of attachment-induced reverse torque. These findings emphasize that attachment design should consider force-couple balance and the resulting moment system to avoid iatrogenic displacement patterns.

The central and lateral incisors responded differently to attachment placement. While certain configurations produced appropriate torque in one incisor but not the other, this discrepancy likely reflects anatomical and biomechanical differences between the two teeth. The lateral incisor has a smaller and more conical root and a reduced PDL surface area compared with the central incisor [19,20], which may increase its susceptibility to tipping and displacement. Because torque expression depends on the moment-to-force ratio generated at the tooth–aligner interface, teeth with smaller roots and reduced PDL support may experience greater rotational displacement under similar force systems. Karsli et al. [15] reported that during the labial movement of palatally positioned lateral incisors, simultaneous placement of attachments on both labial and palatal surfaces provided the most accurate control. In this study, the reverse torque in Group 6 and the minimal root apex displacement (+0.006 mm) indicate that a single labial attachment may be insufficient without adequate anchorage support.

Although the central incisor has a larger root and greater PDL surface area [19,20], these advantages can contribute to adverse effects when attachment placement is unfavorable. In Group 5, minimal crown displacement (+0.002 mm) coupled with substantial root displacement (+0.405 mm) indicates an abnormal center-of-rotation movement. Interestingly, in Group 6, in which only the lateral incisor had an attachment, the central incisor achieved appropriate palatal root torque (75.0%), indicating that attachment placement on adjacent teeth influenced the biomechanical environment of the target teeth. This underscores the importance of considering collective biomechanical environments when designing attachment strategies.

The canine-only attachment configuration demonstrated balanced torque expression for both incisors. This effect may be explained by the preservation of the force-couple system required for palatal root torque while simultaneously providing anchorage support from the canine. Consequently, canine attachments may provide effective anchorage without disrupting the force couple required for anterior torque expression. The canine’s long root and strategic arch-corner position may contribute to improved anchorage stability during anterior torque movements. However, unplanned canine rotation occurred in all groups, with the greatest rotation (−0.538°) observed in Group 3. The canine’s long conical root and arch-corner positions increase its susceptibility to multidirectional forces [7,27], and torque moments transmitted through the CA can induce rotation. Doğruğören et al. [7] suggested that canine attachments can enhance lateral incisor torque efficiency and limit undesired canine rotation, which our findings partially support. Nevertheless, canine rotation remained present even in Group 2 (−0.251°).

Tang et al. [6] emphasized the need for posterior anchorage extending to the first molars when significant anterior torque is planned, and Liu et al. [21] reported the limited effectiveness of canine-only anchorage in certain scenarios. While canine anchorage appeared sufficient for the small 1° torque movement evaluated in this study, the observed rotations indicate that anchorage reinforcement beyond the canine should be considered when moderate or large torque movements are planned (>2–3°). Another consistent finding was the increase in extrusion with a greater number of attachments. Groups with multiple attachments consistently showed greater incisal extrusion than those with fewer or no attachments, with Groups 3 and 4 demonstrating the most pronounced vertical displacement. Although the absolute magnitudes remained below the 2 mm threshold for esthetic perception reported by Kokich et al. [28], these extrusion patterns remain clinically relevant [29]. Several mechanisms may explain this effect. First, our FEA results revealed that multiple attachments increase CA stiffness, which is associated with higher vertical force components [17,18,30]. Second, the palatal force applied near the incisal edge contains an extrusive component that is not perfectly aligned with the tooth’s long axis; Hong et al. [14] also reported similar extrusive tendencies during central incisor torque control. Third, forces from multiple attachments can interact and amplify vertical side effects [31,32]. The greatest extrusion observed in Group 4 suggests cumulative effects. Even a small anterior extrusion can impact smile esthetics, particularly in patients with high smile lines [29], and may complicate occlusal relationships. Clinicians should therefore consider the potential for extrusion when using multiple attachments.

These findings support a “less is more” principle for small torque movements in CA therapy. Rather than maximizing retention with numerous attachments, optimal outcomes can be achieved through strategically placed anchorage attachments that generate favorable force systems without disrupting the force couples necessary for torque expression. However, this concept should be interpreted within the scope of the small-angle torque movements evaluated in this study (approximately 1–2°) and should not be generalized to larger torque corrections or complex multi-stage movements, where the biomechanical role of attachments may differ substantially. This aligns with emerging concepts emphasizing quality and strategic placement over quantity [32]. Future studies should assess whether this approach remains effective for moderate (3–5°) and large (>5°) torque movements.

This study has several limitations. As with all FEAs, the external validity of the present model is inherently limited, and the results should be interpreted as biomechanical approximations under idealized conditions rather than direct representations of clinical outcomes. First, only a small, single-step torque value (1°) was evaluated. Clinical corrections typically involve larger magnitudes and multi-stage sequences, and responses may differ due to cumulative effects, tissue remodeling, and changes in CA fit. Therefore, the present findings should be interpreted primarily as reflecting the initial biomechanical response during early aligner staging rather than the full clinical expression of torque movement. Second, all materials were modeled as linear elastic bodies, although the PDL exhibits nonlinear viscoelastic behavior [33,34], and CA materials undergo time-dependent property changes [35,36]. Third, only horizontal and vertical rectangular attachments were analyzed; other geometries, including ellipsoid, beveled, and optimized designs, may alter force distribution and torque expression [14,37,38]. For example, Hong et al. [14] reported that power ridges provided superior root control compared to attachments, though with distal torsion side effects. Fourth, this study assumed ideal conditions—including perfect alignment, normal periodontal health, complete CA seating, and full patient compliance—whereas clinical movement is influenced by crowding, rotations, periodontal status, occlusal and masticatory forces, and wear compliance [4,39]. In addition, the present model used standardized rectangular attachments to isolate the biomechanical effects of attachment placement while minimizing variability related to attachment geometry. Other attachment shapes, such as ellipsoid or optimized designs, may produce different force distributions and should be investigated in future studies. Fifth, only simultaneous torque application to both incisors was evaluated; alternative staging sequences could produce different responses. Finally, while FEA provides detailed biomechanical insights, the results reflect initial displacement patterns and may not correspond to final clinical outcomes after bone remodeling. Prospective clinical studies using cone-beam computed tomography analysis, digital model superimposition, and long-term follow-up are essential to validate whether the observed biomechanical patterns translate to actual treatment outcomes.

## 5. Conclusions

This FEA study evaluated the effects of attachment placement on palatal root torque control of the maxillary anterior teeth using CAs. The placement of rectangular attachments did not consistently improve torque control, and certain central incisor attachment configurations may produce adverse torque directions under the simulated conditions. These findings should be interpreted as directional biomechanical tendencies rather than direct clinical predictions. The canine-only attachment produced the most balanced torque achievement for both central and lateral incisors. Because central and lateral incisors responded differently to attachment placement, tooth-specific strategies are required. These findings support the use of evidence-based attachment designs to enhance torque control in CA treatment planning.

## Figures and Tables

**Table 1:** Material properties used in this finite element model.

Material	Young’s Modulus (MPa)	Poisson’s Ratio
Alveolar bone	13,700	0.3
Teeth	19,600	0.3
Plastic aligner	528	0.36
Composite attachment	12,500	0.36
Periodontal ligament	0.69	0.45

Note: Material properties were adopted from a previously published study [20].

**Table 2:** Coronal and radicular displacement of the maxillary central and lateral incisors according to attachment configuration.

	Central Incisor’s Edge			Central Incisor’s Root Apex			Lateral Incisor’s Edge			Lateral Incisor’s Root Apex		
Groups	*x*-Axis	*y*-Axis	*z*-Axis	*x*-Axis	*y*-Axis	*z*-Axis	*x*-Axis	*y*-Axis	*z*-Axis	*x*-Axis	*y*-Axis	*z*-Axis
1	0.130	0.130	0.009	0.042	−0.285	−0.087	0.037	0.037	−0.010	−0.165	−0.198	−0.127
2	−0.034	0.058	−0.028	0.043	−0.305	−0.113	0.020	0.019	−0.043	−0.117	−0.242	−0.148
3	0.002	−0.086	−0.184	−0.025	−0.007	−0.165	0.010	−0.071	−0.164	−0.064	−0.021	−0.154
4	0.002	−0.137	−0.204	−0.014	0.107	−0.152	0.010	−0.107	−0.184	−0.054	0.070	−0.124
5	0.042	0.002	−0.079	0.009	0.405	0.011	0.044	−0.031	−0.069	−0.082	−0.189	−0.140
6	−0.013	0.061	−0.030	−0.012	−0.233	−0.096	−0.005	−0.042	−0.110	0.012	0.006	−0.091

Group 1 (control): no attachments on any teeth; Group 2 (C only): attachment on the maxillary canine (#3) only. Group 3 (all anterior): attachments on the central incisor, lateral incisor, and canine. Group 4 (both incisors): Attachments on both central and lateral incisors (#1 and #2). Group 5 (CI only): Attachment on the maxillary central incisor (#1) only. Group 6 (LI only): attachment on the maxillary lateral incisor (#2) only.

**Table 3:** Coronal and radicular displacement of the maxillary canine under different attachment configurations.

	Canine’s Tip			Canine’s Root Apex		
Groups	*x*-Axis	*y*-Axis	*z*-Axis	*x*-Axis	*y*-Axis	*z*-Axis
1	0.054	0.054	0.003	−0.009	0.015	−0.013
2	−0.015	0.032	−0.037	0.035	0.130	0.001
3	0.005	−0.008	−0.098	0.013	0.201	−0.019
4	0.021	−0.022	−0.080	−0.029	0.174	−0.009
5	0.023	−0.044	−0.051	−0.019	0.099	−0.004
6	−0.009	−0.004	−0.055	−0.004	0.145	−0.001

Group 1 (control): no attachments on any teeth. Group 2 (C only): Attachment on the maxillary canine (#3) only. Group 3 (all anterior): attachments on the central incisor, lateral incisor, and canine. Group 4 (both incisors): attachments on both central and lateral incisors (#1 and #2). Group 5 (CI only): attachment on the maxillary central incisor (#1) only. Group 6 (LI only): attachment on the maxillary lateral incisor (#2) only.

**Table 4:** Torque expression of the maxillary central incisor, lateral incisor and canines in each attachment configuration.

Groups	Central Incisor (°)	Lateral Incisor (°)	Canine (°)
1	+1.054	+0.716	+0.102
2	+0.932	+0.762	−0.251
3	−0.202	−0.137	−0.538
4	−0.628	−0.511	−0.502
5	−1.027	+0.471	−0.364
6	+0.750	−0.141	−0.383

Group 1 (control): no attachments on any teeth. Group 2 (C only): attachment on the maxillary canine (#3) only. Group 3 (all anterior): Attachments on the central incisor, lateral incisor, and canine. Group 4 (both incisors): attachments on both central and lateral incisors (#1 and #2). Group 5 (CI only): attachment on the maxillary central incisor (#1) only. Group 6 (LI only): attachment on the maxillary lateral incisor (#2) only.

## Data Availability

The data is contained within the article.

## Abbreviations

The following abbreviations are used in this manuscript:

CAs	Clear Aligners
FEA	Finite Elements Analysis
PDL	Periodontal Ligament
